# Editorial: Aging and work

**DOI:** 10.3389/fpsyg.2025.1673010

**Published:** 2025-08-29

**Authors:** Susana Rubio-Valdehita, Ramón López-Higes, Pedro F. S. Rodrigues, Sara Margarida Fernandes

**Affiliations:** ^1^Department of Social, Work and Differential Psychology, Faculty of Psychology, Complutense University of Madrid, Madrid, Spain; ^2^Department of Experimental Psychology, Cognitive Processes and Speech Therapy, Faculty of Psychology, Complutense University of Madrid, Madrid, Spain; ^3^Department of Psychology and Education, Universidade Portucalense Infante Dom Henrique, Porto, Portugal

**Keywords:** aging, work, healthy aging, cognitive reserve, occupational health, ageism

## 1 Introduction

Population aging represents one of the most profound demographic transformations of the 21st century, with far-reaching implications for global labor markets. As working lives extend and workplaces become increasingly multigenerational, it is critical to understand how older adults adapt to evolving occupational demands. This Research Topic seeks to address these challenges through a multidisciplinary perspective, aiming to promote inclusive, sustainable, and meaningful career trajectories for aging populations.

The Aging and Work Research Topic brings together 14 original contributions that examine these challenges from psychological, organizational, sociological, and public health perspectives. These studies provide robust evidence on how aging intersects with work engagement, health, learning, and quality of life in later adulthood from a multidisciplinary perspective on healthy and sustainable working lives. The findings are organized into four key thematic areas: (1) mental health and emotional wellbeing; (2) workplace design and organizational practices; (3) learning, cognitive function, and career longevity, and (4) social and cultural perspectives on aging and work.

## 2 Mental health and emotional wellbeing

The studies under this theme highlight the multifaceted nature of emotional health in later life. Wang et al. explore longitudinal trajectories of subjective wellbeing, showing how emotional pathways in older adults are linked to anxiety and depression. Lv et al. examine the role of psychological flourishing in the context of mild cognitive impairment, suggesting emotional wellbeing as a protective factor. Onal et al. discuss the impact of nostalgia during leisure time on wellbeing, while Du et al. analyse fatalistic beliefs in disabled elderly populations, highlighting barriers to proactive engagement. Together, these studies underscore the complexity of emotional health in later life and the need for tailored mental health interventions.

## 3 Workplace design and organizational practices

This section emphasizes the strategic role of organizations in supporting longer and healthier careers. Hou et al. demonstrate that age-inclusive Human Resources (HR) practices can enhance career sustainability, with mechanisms such as work–family enrichment and protean career orientation playing a critical role. Hlado et al. propose a model linking perceived work ability among teachers to work-life balance, emphasizing holistic support. Maldonado-Macías et al. explore burnout syndrome among manufacturing workers in Mexico, advocating for ergonomic interventions sensitive to age-related changes. These insights highlight the strategic role of organizations in enabling longer, healthier careers.

## 4 Learning, cognitive function, and career longevity

Lifelong learning emerges as a key factor in prolonging productive working lives. Seeberg et al. find that participation in skills development correlates with improved work ability and delayed retirement, supporting lifelong learning as a key to extended careers. Feng et al. investigate how chronic illnesses interact with cognitive function, emphasizing integrated health monitoring. Zeng et al. report that learning engagement significantly enhances subjective wellbeing among rural “empty nest” elders. Sun et al. add a technological dimension, showing how behavioral factors influence older adults' adoption of smart devices, and calling for initiatives to promote digital inclusion.

## 5 Social and cultural perspectives on aging and work

This theme underscores the impact of cultural and social contexts on older adults' work experiences. Mei et al. study the effects of multidimensional frailty on quality of life among older adults with coronary heart disease, emphasizing the need for functional accommodations. Igreja et al. reveal how older artisans in a UNESCO Creative City report high levels of life satisfaction, suggesting that creative work supports positive aging. Li et al. identify gender disparities in self-rated health among older Chinese workers, advocating for gender-sensitive workforce policies.

## 6 Toward a holistic model of healthy work and aging

**Figure 1:**
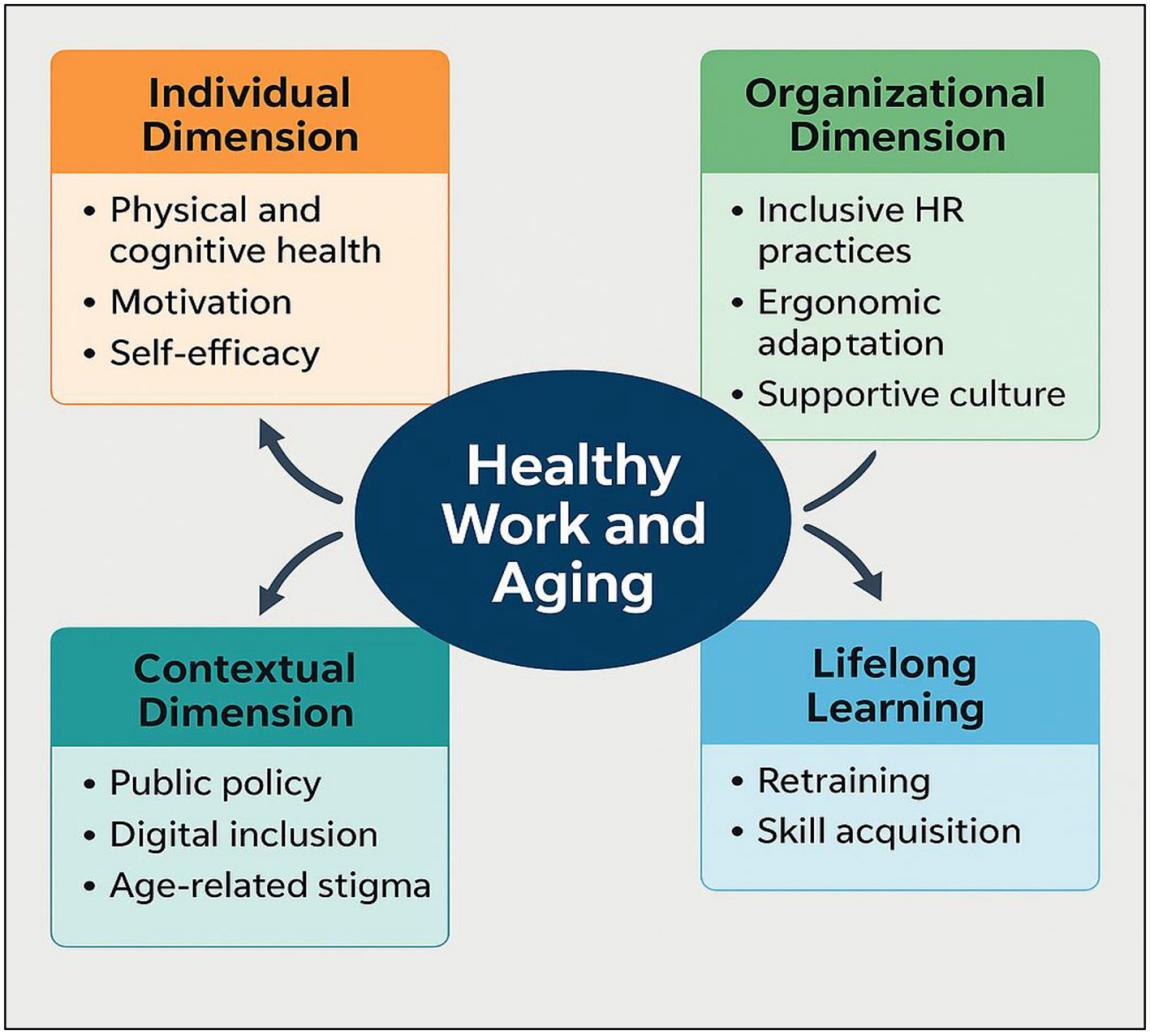
Holistic model of healthy work and aging.

Drawing on the insights provided by the contributions in this Research Topic, we propose a holistic model of healthy work and aging, structured around four interdependent pillars. This model integrates and illustrates the key findings across the collected works, offering a comprehensive framework for understanding and supporting aging in the workplace (see Figure 1):

- Individual dimension: encompasses physical and cognitive health, motivation, and self-efficacy.
- Organizational dimension: focuses on inclusive HR practices, ergonomic adaptation, and age-friendly workplace culture.
- Contextual dimension: includes public policy support, efforts to combat ageism, and initiatives for digital inclusion.
- Lifelong learning: emphasizes ongoing opportunities for retraining, skill renewal, and intellectual engagement at any age.

The figure illustrates a holistic model that integrates four interconnected dimensions essential for promoting healthy and sustainable working lives in the context of aging. At the core lies the Individual Dimension, which encompasses physical health, cognitive functioning, motivation, and self-efficacy—critical personal resources that support work ability in later life. Surrounding this are the Organizational Dimension, which includes age-inclusive human resource practices, ergonomic adaptations, and a workplace culture that values age diversity, and the Contextual Dimension, which involves broader societal and policy-level factors such as public support systems, anti-ageism efforts, and digital inclusion. Finally, the model emphasizes Lifelong Learning as a transversal element that enables older workers to continuously update skills, remain engaged, and adapt to changing work demands. The dynamic interaction among these four dimensions offers a comprehensive framework for understanding and supporting aging workers across diverse occupational and cultural settings.

This model may serve as a strategic guide for organizations, policymakers, and researchers seeking to implement age-inclusive practices and promote wellbeing across the working lifespan.

## 7 Final reflections

The diverse and rich contributions in this *Research Topic* reflect a growing interdisciplinary commitment to reimagining work in the context of aging. Far from viewing age as a limitation, the evidence presented here encourages a redefinition of later-life work as a stage full of potential, personal growth, and meaningful societal contributions.

As we face the demographic challenges of our time, this Research Topic offers both solid empirical foundations and a transformative vision. It calls on researchers, practitioners, and policymakers to design work environments that recognize and value the dignity, capacity, and aspirations of older adults, ultimately fostering a more inclusive, cohesive, and resilient society.

We extend our deepest gratitude to all authors and reviewers for their valuable contributions. Their insights constitute a critical foundation for building healthier, more inclusive, and sustainable workplaces for today's and tomorrow's aging workforce.

